# Diagnostic Utility of Cord Thyroid-Stimulating Hormone (TSH) in Congenital Hypothyroidism and Its Association With Perinatal Factors: A Study From a Tertiary Referral Centre in Hyderabad, India

**DOI:** 10.7759/cureus.73887

**Published:** 2024-11-18

**Authors:** Juwairia Mohammed Fariduddin, Pallavi Chandra Ravula, Venugopal Kura, Sai Kiran D, Suhas Madhukar Chaudhari

**Affiliations:** 1 Laboratory Medicine, Fernandez Foundation, Hyderabad, IND; 2 Obstetrics and Gynaecology, Fernandez Foundation, Hyderabad, IND; 3 Laboratory Services, Fernandez Foundation, Hyderabad, IND; 4 Neonatology, Fernandez Foundation, Hyderabad, IND

**Keywords:** congenital hypothyroidism, cord tsh, newborns, thyroid, universal screening

## Abstract

Introduction

Congenital hypothyroidism (CH) is one of the most common, easily treatable, causes of long-term neurodevelopmental complications in children. The prevalence of CH in India is much higher compared to other countries. Although developed countries have well-established neonatal screening programs, a uniform nationwide screening program at birth is still not established in India. Cord blood thyroid-stimulating hormone (TSH) screening is crucial in diagnosing congenital hypothyroidism even with advancements in expanded newborn screening programs. The aim of this study was to determine the prevalence of elevated cord blood TSH levels and factors associated with it among newborns and to analyze the false positive rates of elevated cord blood TSH levels among the study population and factors associated with it.

Methods

This retrospective cross-sectional study was conducted among 51,251 live newborn babies born at Fernandez Hospital, Hyderabad, India, over a period of five years from January 2019 to December 2023. The data of all the mothers and newborns was retrieved from medical records and details of maternal age, parity, co-morbidities, mode of delivery, gestational age of the newborn at delivery, gender, number of babies, APGAR score, and birth weight were obtained. As per hospital policy, 5 ml of cord blood sample of all newborn babies was collected from the umbilical cord at the time of birth. Samples were analysed for cord TSH batchwise by the enzyme-linked immunosorbent assay (ELISA) method. As per the manufacturer, the normal biological reference intervals are <20 uIU/mL. All the cord TSH values > 20 uIU/ mL were considered abnormal and advised a repeat serum TSH test within 14 days of life. Cord TSH values that were higher than the biological reference range after retesting were considered positive for CH. All the initial and repeat results were retrieved from the neonatal digital database.

Results

Out of 51,251 live newborn babies, 71 (0.14%) were confirmed with a diagnosis of congenital hypothyroidism. The prevalence of CH in our study population was 1.4 per 1000 live births. Anaemia was found to be a statistically significant predictor of true positive cases (p<0.05). In our study, while evaluating risk factors for elevated cord TSH levels, we found that the babies born by vaginal delivery had higher cord TSH levels than babies born by lower segment caesarean section (LSCS) (p<0.001). The other variables considered in the study which include maternal age, BMI, parity of mother, gender of the baby, birth weight, gestation age and APGAR score were not statistically significant for CH.

Conclusion

The prevalence of congenital hypothyroidism was 1.4 in 1000 in our study. Mode of delivery and maternal anaemia had an impact on elevated cord TSH levels. These factors should be considered while interpreting cord TSH values. Cord TSH testing is an effective screening tool for CH. It helps in the early detection of thyroid dysfunction in newborns, enabling timely intervention and treatment to prevent developmental delays and other complications. This study presents evidence supporting the inclusion of cord blood TSH as a predictive factor in CH screening programs.

## Introduction

The thyroid hormones in neonates have a significant impact on brain development [1]. Congenital hypothyroidism (CH) is a medical condition that occurs when an infant is born with an underactive thyroid gland. The causes of CH can be genetic, where there is failure of the thyroid gland to develop properly (dysgenesis) or abnormalities in the production of thyroid hormones. CH is the most common preventable cause of mental retardation [2]. The prevalence of CH in India is 1:1000, much higher when compared to other countries (1:3000-1:4000) [3]. After the introduction of newborn screening programs in developed countries, the prevalence of CH increased from about one in 7000-10,000 to about one in 3000-4000 neonates [4]. The studies published in India reported varying prevalences ranging from one in 727 to one in 2640 [5]. In most newborns, CH does not present with signs and symptoms of thyroid hormone deficiency in the early days of life due to the passage of thyroid hormones across the placenta. By the time the symptoms of thyroid disorders appear in the infant, the thyroid hormone levels are grossly deranged and may cause irreversible sequelae [6,7]. Due to high complications of neonatal hypothyroidism, neonatal cord blood thyroid-stimulating hormone (TSH) screening is performed for timely diagnosis and treatment. Ideally, screening for CH should be done after the TSH surge is over (72 hours of life), but in India, many babies are discharged early, hence cord blood TSH is an easy alternative [8].

During pregnancy, the developing fetus is dependent upon maternal TSH supply for its growth up to 20 weeks of gestation and then starts its own thyroid hormone production. Pregnancy influences thyroid function in various ways. The maternal hypothalamic-pituitary-thyroid (HPT) axis undergoes a series of adjustments. The fetus develops its own HPT axis and the placenta plays an important role in T4 transport and metabolism. Therefore, an integrated three-compartment thyroid model exists during the gestation period. Many countries have developed newborn screening programs to detect CH early. The screening rates are low in India, and an effective social system whereby babies could be reached or recalled after discharge is practically non-existent. There are no national guidelines on screening and follow-up of newborns who are screened positive for CH. Hence, it is extremely important that we have a screening and follow-up protocol in place using cord blood TSH for early diagnosis of CH. It is a simple blood test performed shortly after birth to measure TSH in cord blood samples or that obtained from heel prick samples at three to four days of life. Cord blood TSH has a high sensitivity, and there are high false-positive rates [9]. 

In newborns, several maternal and neonatal factors such as maternal age, maternal anaemia, parity of the mother, mode of delivery, and birth order greatly influence cord TSH levels. Identification of these confounding factors helps in assessing the neonatal thyroid function status. Several studies have been conducted to investigate various maternal and neonatal factors affecting thyroid hormones at birth. However, very few studies have been conducted in India, and those mainly with small sample sizes. Hence, our study aims to analyze perinatal factors affecting neonatal TSH in a large sample size to determine the prevalence of elevated cord blood TSH levels and factors associated with it among newborns and to analyze the false positive rate of elevated cord blood TSH levels among the study population and factors associated with it.

## Materials and methods

This retrospective cross-sectional study was conducted at Fernandez Hospital, Hyderabad, India, over a period of five years from January 2019 to December 2023. The study was started after it was approved by the Ethics Committee of the Fernandez Foundation (approval number: 38-2023). All live newborn babies born at the hospital during the study period were included. A total number of 51,251 newborns were screened for congenital hypothyroidism.

Data collection

The demographic and health information of pregnant women and newborns, including cord TSH and serum TSH data, were obtained from the medical records of Fernandez Hospital. The details such as maternal age, height, weight, parity, gestational age of newborn at delivery, mode of birth, APGAR scores, gender, number of babies, and co-morbidities were collected. 

TSH was measured on dried blood spots by the enzyme-linked immunosorbent assay (ELISA) method (Zentech SA, Liège, Belgium). The measurement of precision of the device was evaluated according to the Clinical and Laboratory Standards Institute (CLSI) document EP05-A3 (%CV 17.4) [10]. In each batch, internal quality controls were run to know the precision of the parameter.

Hospital policy

For the Mother

As per Hospital Protocol, all pregnant mothers were screened for hypothyroidism before 18 weeks of gestation as per standard protocol American Thyroid Association (ATA) 2017 guidelines followed at our institution [11]. The cut-off value for pregnancy screening for hypothyroidism is 0.3-4.0 µIU/ml. For known hypothyroid pregnant mothers, the target values in each trimester are (First Trimester) 0-2-2.5 µIU/, (Second Trimester and Third Trimester) 0.3 - 3.0 µIU/mL

For the Neonate

As per the universal screening methods for early diagnosis of congenital hypothyroidism, cord blood samples of newborn babies were collected by using a BD Vacutainer (Becton, Dickinson and Company, Franklin Lakes, New Jersey, United States) from the umbilical cord at the time of birth of the baby. Cord TSH samples were analyzed by ELISA method for all the babies born at Fernandez Hospital. All the cord TSH values > 20 uIU/mL were considered abnormal and advised repeat serum TSH. The repeat test was done on serum TSH by the chemiluminescence immunoassay (CLIA) method on the Siemens ADVIA XP automated analyzer (Siemens Healthineers AG, Forchheim, Germany) within 14 days of life.

Statistical analysis

Descriptive analysis was carried out by frequency and proportion for categorical variables. The chi-square/fisher exact test was used to test the statistical significance of cross-tabulation between categorical variables. Mann-Whitney U test was used to compare continuous variables between two groups for non-normally distributed variables. Univariate binary logistic regression analysis was performed to test the association between the explanatory variables and outcome; an unadjusted odds ratio along with a 95% CI was presented. A multivariable binary logistic regression model was performed. Adjusted odds ratio along with 95% CI was presented. P value <0.05 was considered statistically significant. RStudio desktop version 2023.03.0+ 386 (RStudio, PBC, Boston, Massachusetts, United States) was used for statistical analysis.

## Results

**Figure 1:**
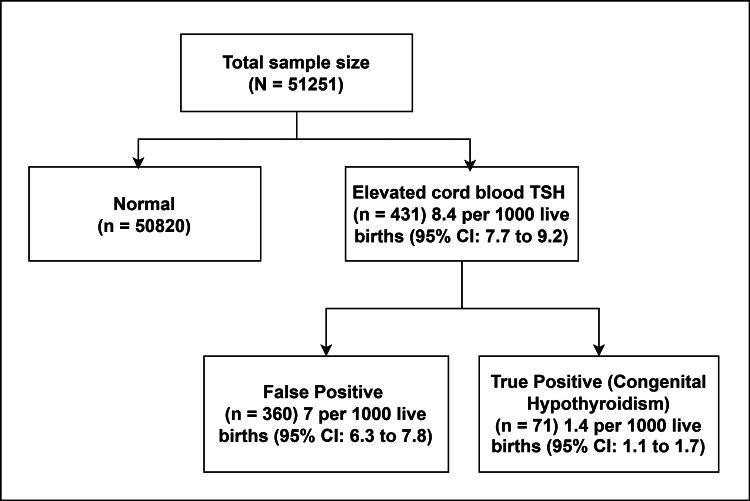
Flowchart of the study population and sample size for analysis of factors associated with cord TSH

A total of 51,251 live-born babies were included in the final analysis during the study period. Among these 51,251 babies, 431 babies had elevated cord TSH levels >20 IU/mL. Out of these 431 babies, 32 were lost to follow-up. In the remaining 399 babies, on retesting, 71 babies were diagnosed to have CH. The incidence of CH in our study was 1.4 per 1000 live births. The incidence of elevated cord blood TSH levels was 8.4 per 1000 live births. The false positive rate of elevated cord blood TSH levels was 7 per 1000 live births (Figure 1).

**Figure 2:**
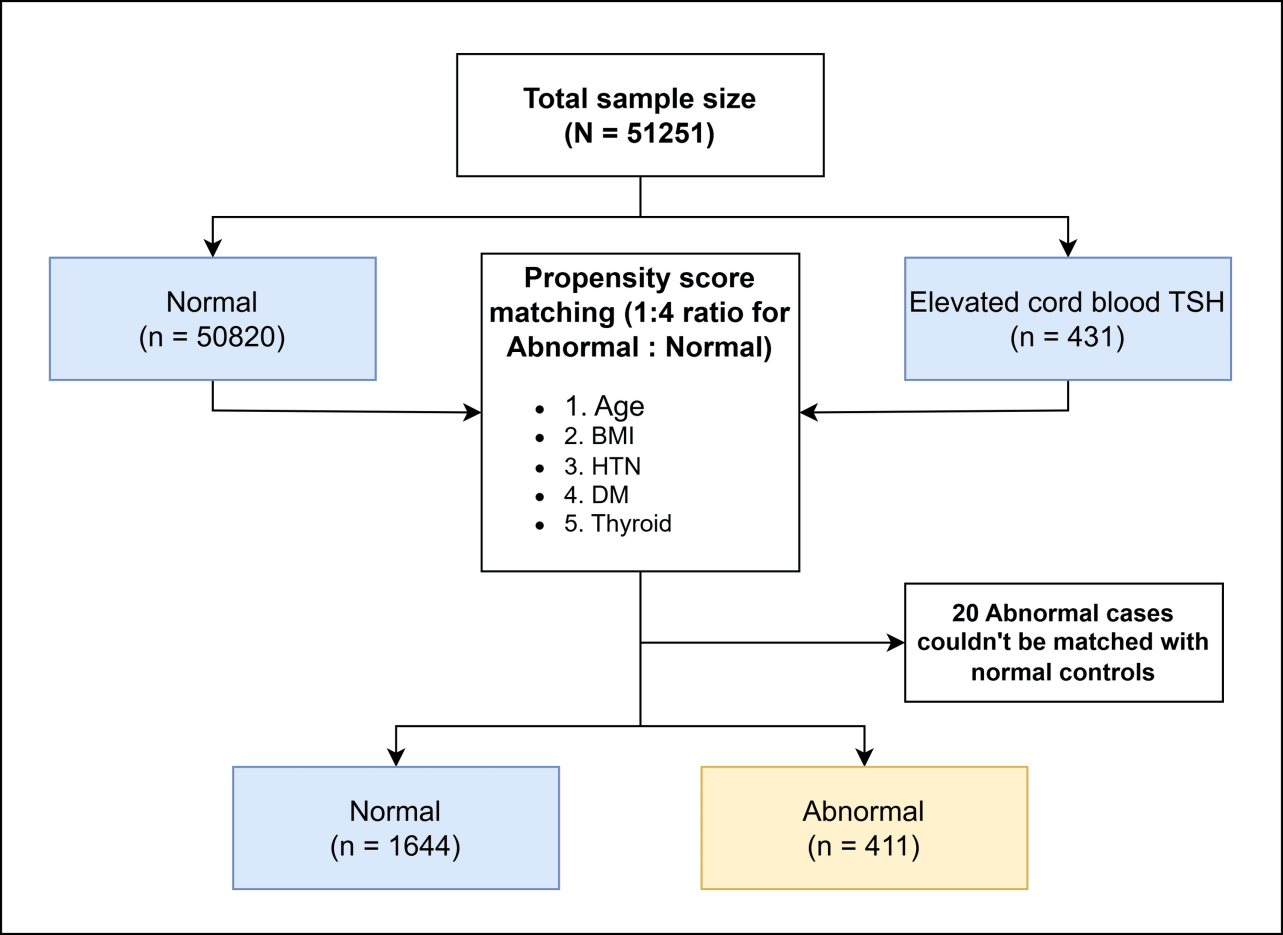
Flowchart depicting propensity score matching BMI: body mass index; HTN: hypertension; DM: diabetes mellitus; TSH: thyroid-stimulating hormone

Among 431 newborns with elevated cord blood TSH, 32 were lost to follow-up and were considered negative for eventual CH as this number is least likely to impact the overall calculations considering the large sample size. To analyze the factors associated with different categories of thyroid status, we performed propensity score matching for potential confounders (age, BMI, hypertension, diabetes mellitus, and thyroid status of mother) with an elevated TSH group and normal group ratio of 1:4. During the matching, 11 cases were excluded because they couldn’t be matched with appropriate controls. The final analysis included a total of 2055 newborns (411 with elevated cord blood TSH and 1644 with normal cord blood TSH) (Figure 2).

We analyzed the association between elevated neonatal TSH and maternal age, BMI, comorbidities, mode of delivery, mode of conception, and parity. The neonatal factors analyzed were gestational age, gender of the baby, number of fetuses, birth weight, and APGAR. None of the maternal and neonatal factors showed statistically significant differences between the babies born with normal and elevated cord TSH levels except the mode of delivery (Table 1).

**Table 1:** Comparison of effects of maternal and neonatal factors on cord TSH among the study groups ^*^ Chi-square test, ^#^ Mann whitney u test TSH: thyroid-stimulating hormone

Parameters	Normal (Propensity matched controls) (n=1644), n (%)	Abnormal (Elevated cord blood TSH levels) (n=411), n (%)	P value
Maternal Factors			
Age Groups (years)			
17-25	375 (22.8%)	106 (25.8%)	0.317*
26-35	1186 (72.1%)	281 (68.4%)	
>35	83 (5%)	24 (5.8%)	
Body mass index category			
18.5-24.9	648 (39.4%)	160 (38.9%)	0.867*
25-29.9	670 (40.8%)	173 (42.1%)	
≥ 30	326 (19.8%)	78 (19%)	
Comorbidities			
Hypertension	197 (12%)	48 (11.7%)	0.865*
Diabetes mellitus	448 (27.3%)	113 (27.5%)	0.921*
Thyroid	426 (25.9%)	123 (29.9%)	0.100*
Pre-eclampsia	63 (3.8%)	13 (3.2%)	0.520*
Renal	19 (1.2%)	3 (0.7%)	0.597*
Abnormal haemoglobin variants	40 (2.4%)	7 (1.7%)	0.376*
Anemia	354 (21.5%)	105 (25.5%)	0.08*
Parity			
<2	1443 (87.8%)	363 (88.3%)	0.761*
≥2	201 (12.2%)	48 (11.7%)	
Mode of Conception			
Spontaneous	1489 (90.6%)	388 (94.4%)	0.067*
Intrauterine insemination (IUI)	20 (1.2%)	2 (0.5%)	
In vitro fertilization (IVF)	81 (4.9%)	10 (2.4%)	
Ovulation induction	54 (3.3%)	11 (2.7%)	
Mode of Delivery			
Spontaneous vaginal birth	659 (40.1%)	221 (53.8%)	<0.001*
Assisted vaginal birth	188 (11.4%)	65 (15.8%)	
L*ower segment caesarian section*	797 (48.5%)	125 (30.4%)	
Neonatal Factors			
Gestation age			
Term	1366 (83.1%)	363 (88.3%)	0.073*
Extreme Preterm	34 (2.1%)	7 (1.7%)	
Preterm	212 (12.9%)	35 (8.5%)	
Post Term	32 (1.9%)	6 (1.5%)	
Gender			
Male	818 (49.8%)	198 (48.2%)	0.566*
Female	826 (50.2%)	213 (51.8%)	
Number of Foetus			
Singleton	1548 (94.2%)	397 (96.6%)	0.05*
Multi-fetal pregnancy	96 (5.8%)	14 (3.4%)	
APGAR 5 minutes			
<7	15 (0.9%)	7 (1.7%)	0.179*
≥7	1629 (99.1%)	404 (98.3%)	
Birth weight (Median, IQR)	2.94 (2.60, 3.24)	2.94 (2.64, 3.23)	0.883#

None of the maternal and neonatal factors were found to be statistically significant with congenital hypothyroidism (true positive) when compared to babies with normal cord TSH levels (Table 2).

**Table 2:** Effects of maternal and neonatal factors associated with congenital hypothyroidism ^*^ Chi-square test, ^#^ Mann Whitney U test

Parameters	Normal (Propensity matched controls) (n=1644), n (%)	True Positive (True congenital hypothyroidism) (n=68), n (%)	P value
Maternal Factors			
Age Groups (years)			
17-5	375 (22.8%)	15 (22.1%)	0.958*
26-35	1186 (72.1%)	50 (73.5%)	
>35	83 (5%)	3 (4.4%)	
Body Mass Index category			
18.5-24.9	648 (39.4%)	26 (38.2%)	0.978*
25-29.9	670 (40.8%)	28 (41.2%)	
≥30	326 (19.8%)	14 (20.6%)	
Comorbidities			
Hypertension	197 (12%)	6 (8.8%)	0.430*
Diabetes mellitus	448 (27.3%)	21 (30.9%)	0.511*
Thyroid	426 (25.9%)	21 (30.9%)	0.361*
Pre-eclampsia	63 (3.8%)	4 (5.9%)	0.337*
Renal	19 (1.2%)	1 (1.5%)	0.557*
Abnormal haemoglobin variants	40 (2.4%)	1 (1.5%)	1*
Anemia	354 (21.5%)	9 (13.2%)	0.101*
Mode of Conception			
Spontaneous	1489 (90.6%)	64 (94.1%)	0.654*
Intrauterine Insemination (IUI)	20 (1.2%)	0 (0%)	
In vitro fertilization (IVF)	81 (4.9%)	3 (4.4%)	
Ovulation induction	54 (3.3%)	1 (1.5%)	
Parity			
<2	1443 (87.8%)	64 (94.1%)	0.114*
≥2	201 (12.2%)	4 (5.9%)	
Mode of Delivery			
Spontaneous vaginal birth	659 (40.1%)	35 (51.5%)	0.166*
Assisted vaginal birth	188 (11.4%)	7 (10.3%)	
Lower segment caesarian section	797 (48.5%)	26 (38.2%)	
Neonatal Factors			
Gestation age			
Term	1366 (83.1%)	59 (86.8%)	0.522*
Extreme Preterm	34 (2.1%)	2 (2.9%)	
Preterm	212 (12.9%)	5 (7.4%)	
Post Term	32 (1.9%)	2 (2.9%)	
Gender			
Male	818 (49.8%)	30 (44.1%)	0.362*
Female	826 (50.2%)	38 (55.9%)	
Number of Foetus			
Singleton	1548 (94.2%)	65 (95.6%)	0.795*
Multi-fetal pregnancy	96 (5.8%)	3 (4.4%)	
APGAR 5 minutes			
<7	15 (0.9%)	0 (0%)	1*
≥7	1629 (99.1%)	68 (100%)	
Birth weight	2.94 (2.60, 3.24)	2.88 (2.66, 3.19)	0.663#

On univariate analysis, only the mode of delivery had a statistically significant association with elevated cord blood TSH levels. In multivariate analysis, after adjusting for the effect of gestational age at delivery and mode of conception, the odds of elevated TSH level were higher in spontaneous vaginal birth (aOR=2.027, 95%CI 1.58 to 2.6, P<0.001) and assisted vaginal birth (aOR= 2.11, 95%CI 1.49-2.98, P<0.001) as compared to babies born by LSCS. None of the other maternal and neonatal parameters showed a statistically significant association (Table 3).

**Table 3:** Univariate and multivariable binary logistic regression to predict elevated cord blood TSH levels TSH: thyroid-stimulating hormone

Parameters	Unadjusted OR (95% CI)	P value	Adjusted OR (95% CI)	P value
Maternal parameters				
Age Groups (years)				
17-25	(Ref)		-	
26-35	0.838 (0.652-1.078)	0.169	-	-
>35	1.023 (0.619-1.691)	0.929		
Body Mass Index category				
18.5-24.9	(Ref)		-	
25-29.9	1.046 (0.822-1.330)	0.716	-	-
≥30	0.969 (0.717-1.310)	0.838		
Comorbidities				
Hypertension	0.971 (0.694-1.359)	0.865	-	-
Diabetes mellitus	1.012 (0.795-1.290)	0.921	-	-
Thyroid	1.221 (0.962-1.550)	0.1	-	-
Pre-eclampisa	0.820 (0.447-1.504)	0.521	-	-
Renal	0.629 (0.185-2.135)	0.457	-	-
Abnormal haemoglobin variants	0.695 (0.309-1.562)	0.378	-	-
Anemia	1.250 (0.973-1.607)	0.081	-	-
Mode of Conception				
Spontaneous	(Ref)		(Ref)	
Intrauterine Insemination (IUI)	0.384 (0.089-1.649)	0.198	0.489 (0.112-2.130)	0.341
In vitro fertilization (IVF)	0.474 (0.243-0.923)	0.028	0.673 (0.334-1.354)	0.267
Ovulation induction	0.782 (0.405-1.509)	0.463	0.817 (0.420-1.589)	0.551
Parity				
<2	(Ref)		-	-
≥2	0.949 (0.679-1.327)	0.761	-	
Mode of birth				
Cesarean section	(Ref)		(Ref)	
Spontaneous vaginal birth	2.138 (1.678-2.725)	<0.001	2.027 (1.580-2.600)	<0.001
Assisted vaginal birth	2.204 (1.570-3.095)	<0.001	2.113 (1.495-2.988)	<0.001
Neonatal Parameters				
Gestation Age Category				
Term	(Ref)		(Ref)	
Extreme Preterm	0.775 (0.341-1.762)	0.543	1.157 (0.492-2.721)	0.738
Preterm	0.621 (0.427-0.905)	0.013	0.827 (0.559-1.223)	0.341
Post Term	0.706 (0.293-1.700)	0.437	0.716 (0.294-1.741)	0.461
Gender				
Male	(Ref)		-	-
Female	1.065 (0.858-1.323)	0.566	-	
Number of Fetuses				
Singleton	(Ref)		-	-
Multi-fetal pregnancy	0.569 (0.321-1.007)	0.053	-	
APGAR 5 minutes				
<7	(Ref)		-	-
≥7	0.531 (0.215-1.312)	0.17	-	
Birth weight	1.048 (0.856-1.283)	0.649	-	-

**Figure 3:**
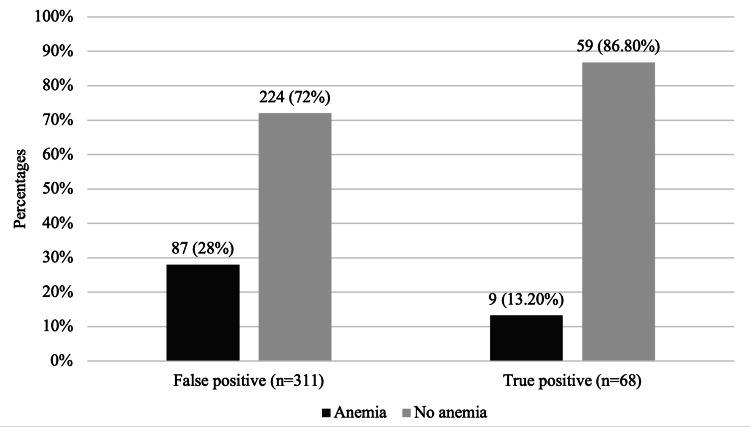
Association between anaemia and false positive cord blood TSH levels P Value: 0.011 Statistical test used: Chi-square test TSH: thyroid-stimulating hormone

According to univariate and multivariate analysis, none of the maternal and neonatal parameters showed a statistically significant association with congenital hypothyroidism (Table 4). Maternal anaemia was associated with false-positive elevated cord blood TSH levels. Among false positives, 87 (28%) were anaemic, as compared to nine (13.2%) in true positives (P=0.011) OR 2.54 (1.21-5.35) (Figure 3).

**Table 4:** Univariate and multivariate binary logistic regression to predict true positive cases

Parameters	Unadjusted OR (95% CI)	P value	Adjusted OR (95% CI)	P value
Maternal Factors				
Age Groups (years)				
17-25	(Ref)		-	
26-35	1.054 (0.585-1.899)	0.861	-	-
>35	0.904 (0.256-3.193)	0.875		
BMI category				
18.5-24.9	(Ref)		-	
25-29.9	1.042 (0.604-1.796)	0.883	-	-
≥30	1.070 (0.551-2.078)	0.841		
Comorbidities				
Hypertension	0.711 (0.303-1.665)	0.432	-	-
Diabetes mellitus	1.193 (0.705-2.018)	0.511	-	-
Thyroid disorders	1.277 (0.755-2.162)	0.362	-	-
Pre-eclampisa	1.568 (0.554-4.442)	0.397	-	-
Renal disorders	1.277 (0.168-9.677)	0.813	-	-
Abnormal haemoglobin variants	0.599 (0.081-14.419	0.377	-	-
Anemia	0.556 (0.273-1.132)	0.106	-	-
Mode of Conception				
Spontaneous	(Ref)		(Ref)	
Intrauterine insemination (IUI)	0.00 (0.00-inf)	0.998	0.00 (0.00-inf)	0.998
In vitro fertilization (IVF)	0.862 (0.265-2.802)	0.805	1.117 (0.312-3.995)	0.865
Ovulation induction	0.431 (0.059-3.164)	0.408	0.448 (0.061-3.307)	0.431
Parity				
<2	(Ref)		-	-
≥2	0.449 (0.162-1.245)	0.124	-	
Mode of Birth				
Cesarean section	(Ref)		(Ref)	
Spontaneous vaginal birth	1.628 (0.970-2.733)	0.065	1.590 (0.934-2.708)	0.088
Assisted vaginal birth	1.141 (0.488-2.669)	0.76	1.073 (0.453-2.540)	0.873
Neonatal Parameters				
Gestation age at birth				
Term	(Ref)		(Ref)	
Extreme preterm	1.362 (0.320-5.804)	0.676	1.492 (0.324-6.878)	0.608
Preterm	0.546 (0.217-1.376)	0.199	0.612 (0.237-1.582)	0.311
Post term	1.447 (0.339-6.182)	0.618	1.607 (0.372-6.942)	0.525
Gender				
Male	(Ref)		-	-
Female	1.254 (0.770-2.044)	0.363	-	
Number of Foetus				
Singleton	(Ref)		-	-
Multi-fetal pregnancy	0.744 (0.230-2.411)	0.622	-	
APGAR 5 minutes				
<7	(Ref)		-	-
≥7	6744 (0.00-inf)	0.999	-	
Birth weight	0.951 (0.612-1.479)	0.824	-	-

## Discussion

Congenital hypothyroidism when left untreated has serious sequelae such as neurodevelopmental delay and intellectual disability in the newborn. Considering the high birth rates in India, universal screening of newborns at birth for CH by estimating cord TSH levels is an easy and practical method to detect and treat the condition in a timely manner. Neonatal screening involves various methods like measuring the cord blood TSH or neonatal heel prick within three to four days of life.

The postnatal screen using a heel prick after 48-72 hours of life is adopted by most countries with universal newborn screening programmes. It has the limitations of needing to follow up if discharged and sampling technique requiring expertise. In LMIC countries, compliance with neonatal screening is poor due to the absence of guidelines, the high burden of births, and early discharges. Thus, at birth, cord TSH offers a unique opportunity to screen newborns for CH. Cord blood investigation is a well-established technique for neonatal investigations [12]. However, cord TSH screening is not well established, and diagnostic utility is not well established, especially in LMIC settings. Kaul et al. report it to have high sensitivity and high false-positive rates [9]. In the current study, as per the manufacturer (Zentech SA)'s kit insert, diagnostic specificity was 99.7%, and the positive predictive value was 0.039. This might lead to high recall rates and thus costs. 

To the best of our knowledge, this is one of the first studies from India done on a large population cohort. The present study population was part of a well-established cord TSH screening programme at our institute over the last five years. In this population cohort, the incidence of CH was 1.4 per 1000 liveborn babies, which is comparable to other studies reported from India. The reported incidence varies from one in 97 to three in 1000, and a recent meta-analysis reported an overall CH prevalence of 0.97 per thousand (1:1031) that ranged from <1:4057 to 1:23 [13,14,8].

The cord TSH cutoff of > 20 IU/mL for considering screen positive has been reported in an earlier study done by Ravi et al. on 2916 neonates, which concluded that a cutoff cord blood TSH value > 20 IU/mL can be used for screening congenital hypothyroidism [8]. There are several perinatal factors that may affect cord TSH levels [15,16]. In the current study, we aimed to find the correlation between perinatal factors such as maternal age, BMI, parity, maternal comorbidities, number of fetuses, mode of birth, gestational age at birth, gender of the baby, weight of the baby and APGAR score with elevated cord TSH levels.

Postnatal surge in TSH is very common in newborns, which is due to adrenergic stimulation following cold stress. In the present study, on analyzing the perinatal factors affecting CH, CH was not associated with maternal demographic factors such as age, BMI, parity, or mode of conception, which is similar to other studies [14,16,17]. Few studies showed a higher incidence of CH in primigravida when compared to multiparous women [14,18]. On analyzing maternal medical conditions like gestational diabetes, gestational hypertension, and maternal hypothyroid status, our study did not find a statistically significant correlation between maternal hypothyroid status and CH.

Few studies showed the correlation of maternal hypothyroid status with CH [19,20]. According to univariate analysis, anaemia was found to be a statistically significant predictor of true positive cases (p <0.05). Cases with anaemia were 60.7% (unadjusted OR: 0.393; 95%CI: 0.187 to 0.826) less likely to be true positive as compared to cases without anaemia. One possible mechanism is that iron deficiency impairs the heme-dependent TPO enzyme, which limits the synthesis of thyroid hormones. Cord blood TSH levels are influenced by maternal anaemia. A study done by Varma et.al showed that cord TSH levels had a significant inverse relationship with maternal haemoglobin levels which is similar to our findings [21]. The biological plausibility for increased cord TSH among mothers with anaemia is not known and requires further evaluation. On analyzing the mode of birth, babies born vaginally had more incidence of CH than those delivered by caesarean section, similar to other studies [15,19,22]. The reason behind this was proposed to be due to the release of a high amount of catecholamines during vaginal birth when compared to cesarean sections [18]. The gender of the baby had no significant effect between the two groups of normal and elevated cord TSH levels in our study, in contrast to the studies that showed a male gender preponderance for CH [15,18]. The birth weight of the baby did not have any correlation with CH similar to other studies [18,23,24]. Among the 411 babies with elevated cord TSH levels, 363 (88.3%) babies were term babies, whereas 35 (8.5%) were preterm and seven (1.7%) were extreme preterm. Raised TSH levels were seen in term babies who had well-developed hypothalamic-pituitary axis. Studies showed no significant difference in the gestational age of the baby and CH [14,16]. The strength of the study is that it was conducted with a large population cohort and data from a well-established screening programme. The limitation of the study is its retrospective nature. We need a prospective large collaborative study with other peripheral centres to know the burden of the condition. The other limitation is the lack of data on a few cases not picked up on cord TSH and subsequently diagnosed CH (false negatives). 

## Conclusions

Cord TSH-based screening is a feasible and inexpensive test. The cord TSH screening needs to be interpreted carefully in vaginal births, maternal anaemia, and preterm births. There is a need for multicentre data involving longer follow-up and evaluating the cost impact of this screening strategy, revealing that increased levels of cord TSH are due to perinatal stress during vaginal births. We highly recommend that universal CH screening should be included in India's national health programs as early diagnosis and treatment result in good outcomes.
